# Cytotoxic constituents and a new hydroxycinnamic acid derivative from *Leontodon saxatilis* (Asteraceae, Cichorieae)†

**DOI:** 10.1039/d0ra10973h

**Published:** 2021-03-10

**Authors:** Serhat Sezai Ҫiҫek, Johanna Willer, Francesca Preziuso, Frank Sönnichsen, Richard Greil, Ulrich Girreser, Christian Zidorn, Karin Jöhrer

**Affiliations:** Department of Pharmaceutical Biology, Kiel University Gutenbergstraße 76 24118 Kiel Germany scicek@pharmazie.uni-kiel.de; Department of Pharmacy, University “G. d'Annunzio” of Chieti-Pescara Via dei Verstini 31 66100 Chieti Scalo (CH) Italy; Otto Diels Institute for Organic Chemistry, Kiel University Otto-Hahn-Platz 4 Kiel Germany; Tyrolean Cancer Research Institute Innrain 66 6020 Innsbruck Austria; Paracelsus Medical University Salzburg, Department of Internal Medicine III, Salzburg Cancer Research Institute-Laboratory for Immunological and Molecular Cancer Research Müllner Hauptstraße 48 5020 Salzburg Austria; Department of Pharmaceutical and Medicinal Chemistry, Kiel University Gutenbergstraße 76 24118 Kiel Germany

## Abstract

In our ongoing research for the discovery of new constituents with antimyeloma activity, we investigated 15 compounds present in the aerial parts of *Leontodon saxatilis* for their cytotoxic potential against NCI-H929, U266, and OPM2 cell lines. One of the isolated compounds displayed a new natural product and was identified as 5-feruloyl-2α-hydroxyquinic acid after LC-MS and NMR experiments. Of the remaining compounds, cichoric acid and three flavone glycosides, apigenin 4′-*O*-β-d-glucoside, luteolin 7-*O*-β-d-glucoside and luteolin 4′-*O*-β-d-glucoside, showed moderate cytotoxic activity, whereas the effects of two aglyones apigenin and luteolin were more pronounced. Though the cytotoxic potential of the two aglycones (against other cell lines) was reported in various studies, our work moreover showed that cooccurrence of these two compounds with similar components of lower activity led to comparable results and at the same time minimized the damage of healthy fibroblast cells. Thus, our work could be a starting point for additional studies on the synergistic effect of similar components against myeloma cell lines.

## Abstract 2

Phytochemical investigation of the aerial parts of *Leontodon saxatilis* yielded six compounds with antimyeloma activity as well as crepidiaside A as a chemophenetic marker and 5-feruloyl-2α-hydroxyquinic acid as a new hydroxycinnamic acid derivative.

## Graphical abstract

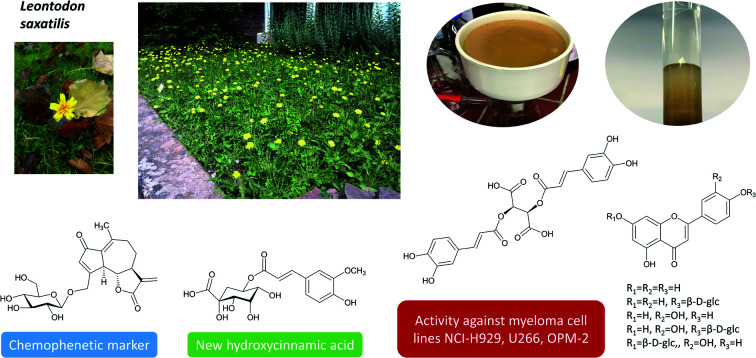

## 1. Introduction

*Leontodon saxatilis* Lam., Asteraceae, is a species native to warm and temperate regions of Europe and Northern Africa.^1–3^ It has also been introduced into other regions such as California, Chile, New Zealand, and Southern Australia.^4,5^ Because of its high invasiveness, *L. saxatilis* was the subject of several ecological studies investigating its ecotypic differentiation, its ability to produce two morphologically distinct fruits and its seed production under elevated carbon dioxide levels.^1,4–7^

Regarding the constituent pattern of *L. saxatilis*, so far only five constituents have been identified by LC-MS analysis, including chlorogenic acid and 3,5-dicaffeoylquinic acid.^8^ Related species of the same genus have been found to contain additional hydroxycinnamic acid derivatives, together with flavonoids of the luteolin-type, and sesquiterpene lactones.^9–11^ In order to further assess the metabolome of *L. saxatilis*, we decided to study the phytochemical composition of this interesting species. Moreover, we discovered activity for a crude extract against two myeloma cell lines in a cytotoxicity screening, which also led to the previously reported identification of antimyeloma constituents in *Turnera diffusa* and *Actaea racemosa*.^12,13^

Multiple myeloma is a plasma cell malignancy, which is mainly restricted to the bone marrow.^14^ The current standard treatment includes immunomodulatory drugs and proteasome inhibitors, such as bortezomib.^15^ But also natural products have been found to inhibit proteasome catalytic activities, such as the flavones apigenin, chrysin, and luteolin.^16^ Moreover, sesquiterpenes are known to exhibit strong cytotoxic effects, *e.g.* β-caryophyllene or the lactone cnicin, which showed pronounced activity against myeloma cells.^17,18^ Since multiple myeloma is still incurable, novel therapeutics are urgently needed and natural products might provide a base for new medications.

## 2. Materials and methods

### 2.1. General experimental procedures

Thin layer chromatography was performed using ethyl acetate–water–acetic acid–formic acid (20 : 5.4 : 2.2 : 2.2) as the eluent and vanillin sulfuric acid as the spraying reagent. Preparative medium pressure liquid chromatography (MPLC) was carried out with a Büchi PrepChrom C-700 chromatograph using a PrepChrom C18 column (250 × 30.0 mm, 15 m particle size, Büchi Labortechnik GmbH, Essen, Germany). UHPLC analyses were performed on a VWR-Hitachi Chromaster Ultra RS equipped with a 6170 binary pump, 6270 autosampler, 6310 column oven, 6430 DAD, and VWR 100 evaporative light scattering detector (VWR International GmbH, Darmstadt, Germany). LC-MS analyses were carried out on a Shimadzu Nexera 2 liquid chromatograph connected to an LC-MS triple quadrupole mass spectrometer using electrospray ionization (Shimadzu, Kyoto, Japan). A Phenomenex Luna Omega C18 column and a Luna Omega C18 polar column (both with 100 × 2.1 mm, 1.6 μm particle size, Phenomenex, Aschaffenburg, Germany) were employed for the analysis of extracts, fractions, and pure compounds. HR-ESI-MS spectra were recorded on a Q-Exactive Plus spectrometer (Thermo Scientific, Bremen, Germany) and optical rotation was measured on a PerkinElmer 241 Polarimeter (PerkinElmer, Rodgau, Germany). NMR spectra were recorded on a Bruker Avance III 400 NMR spectrometer operating at 400 MHz for the proton channel and 100 MHz for the ^13^C channel with a 5 mm PABBO broad band probe with a *z* gradient unit at 293 K (Bruker BioSpin GmbH, Rheinstetten, Germany). Reference values were 3.31 (^1^H) and 49.15 ppm (^13^C) for methanol as well as 2.50 (^1^H) and 39.51 ppm (^13^C) for DMSO, respectively.

### 2.2. Chemical reagents

LC-MS grade formic acid, Diaion HP-20, and Sephadex LH-20 were purchased from Sigma Aldrich Co., St. Louis, MO, USA. Silica gel (40–63 μm) for column chromatography, TLC plates (silica gel 60 F254), acetonitrile and water (both of LC-MS grade), gradient grade methanol, and other (analytical grade) solvents were obtained from VWR International GmbH, Darmstadt, Germany. Water used for isolation was doubly distilled in-house. Apigenin (14, Lot A002/02-08) and luteolin (15, Lot L007/02-08) were obtained from TransMIT, Marburg, Germany. Solid phase extraction (SPE) columns (Chromabond HR-XA 3 mL/500 mg) were obtained from Macherey-Nagel GmbH and Co. KG, Düren, Germany. Dimethyl sulfoxide-*d*_6_ (99.80%, Lot S1051, Batch 0119E) and methanol-*d*_4_ (99.80%, Lot P3021, Batch 1016B) for NMR spectroscopy were purchased from Euriso-top GmbH, Saarbrücken, Germany, and conventional 5 mm NMR sample tubes were obtained from Rototec-Spintec GmbH, Griesheim, Germany.

### 2.3. Plant material

*L. saxatilis* samples for initial cytotoxicity screenings were collected and identified in Kiel-Wik on August 18, 2016 in front of the general directorate for waterways and shipping, N 54°21′12.6′′, E 10°8′24.2′′, by S. S. Ҫiҫek. A voucher specimen is deposited in the hebarium of SSC (specimen number: SSC-2016_006). In the following two years, *L. saxatilis* was cultivated in the Medicinal Plant Garden of the Pharmaceutical Institute in Kiel using seeds obtained from the Botanical Garden of the University of Constance (Germany). Voucher specimens of plant material from both years are also deposited in the herbarium of SSC (numbers: SSC-2017_027 and SSC-2018_012).

### 2.4. Extraction and isolation

Dried and ground aerial parts of *L. saxatilis* (1.00 kg) were extracted five times with 2 L of acetone (70% v/v) using ultra-sonification for 15 minutes followed by maceration for at least 12 hours each. After evaporation of the acetone, the remaining aqueous solution was repeatedly extracted with ethyl acetate giving 38.6 g of ethyl acetate extract. The remaining water layer was further extracted with *n*-butanol to give another 17.6 g of *n*-butanol extract.

For further separation, the ethyl acetate extract was subjected to vacuum liquid chromatography, using silica gel as stationary phase. After loading the extract onto the stationary phase, the sample was subsequently eluted with *n*-hexane (800 mL), dichloromethane (600 mL), acetone (600 mL), and methanol (600 mL), respectively. After evaporation of the solvents, fractions of 8.40 g (*n*-hexane), 9.24 g (dichloromethane), 13.05 g (acetone), and 3.46 g (methanol) were obtained.

The *n*-butanol extract was also subjected to vacuum liquid chromatography. This time Diaion HP-20 material was used as stationary phase and the following solvents were applied: water (800 mL), methanol 25% (400 mL), methanol 50% (600 mL), methanol 75% (1 L) and methanol (600 mL) to give fractions of 5.98 g (water), 1.91 (methanol 25%), 3.81 g (methanol 50%), 3.31 g (methanol 75%), and 0.95 g (methanol) respectively.

The fraction obtained from methanol 50% was subjected to Sephadex LH-20 chromatography (100 × 3 cm, in methanol) to give 7 subfractions. Subfraction 2 (398 mg) was subjected to preparative MPLC using water (A) and methanol (B) with a flow rate of 27 mL min^−1^ and the following gradient: 5% B to 35% B in 60 min, and to 95% B in 90 min. Of the resulting 6 subfractions, subfraction 2 yielded 1.5 mg of compound 1, subfraction 4 yielded 7.6 mg of compound 2, subfraction 6 yielded 0.5 mg of compound 3 and subfraction 8 afforded 14.5 mg of compound 4.

The fraction obtained from methanol 75% was subjected to the same Sephadex LH-20 chromatography column as above to give 11 subfractions. Subfraction 1 yielded compounds 5 and 6 after separation by anion exchange solid phase extraction (AX-SPE). Subfraction 2 yielded 28.7 mg of compound 7. Subfractions 4 yielded 21.0 mg of compound 8 and 24.5 mg of compound 9 after AX-SPE. Subfraction 6 yielded 6.1 mg of compound 10 and 9.3 mg of compound 11 after AX-SPE. Subfraction 8 yielded 19.6 mg of compound 12 and subfraction 7 afforded 6.3 mg of compound 13 after purification AX-SPE.

**Fig. 1:**
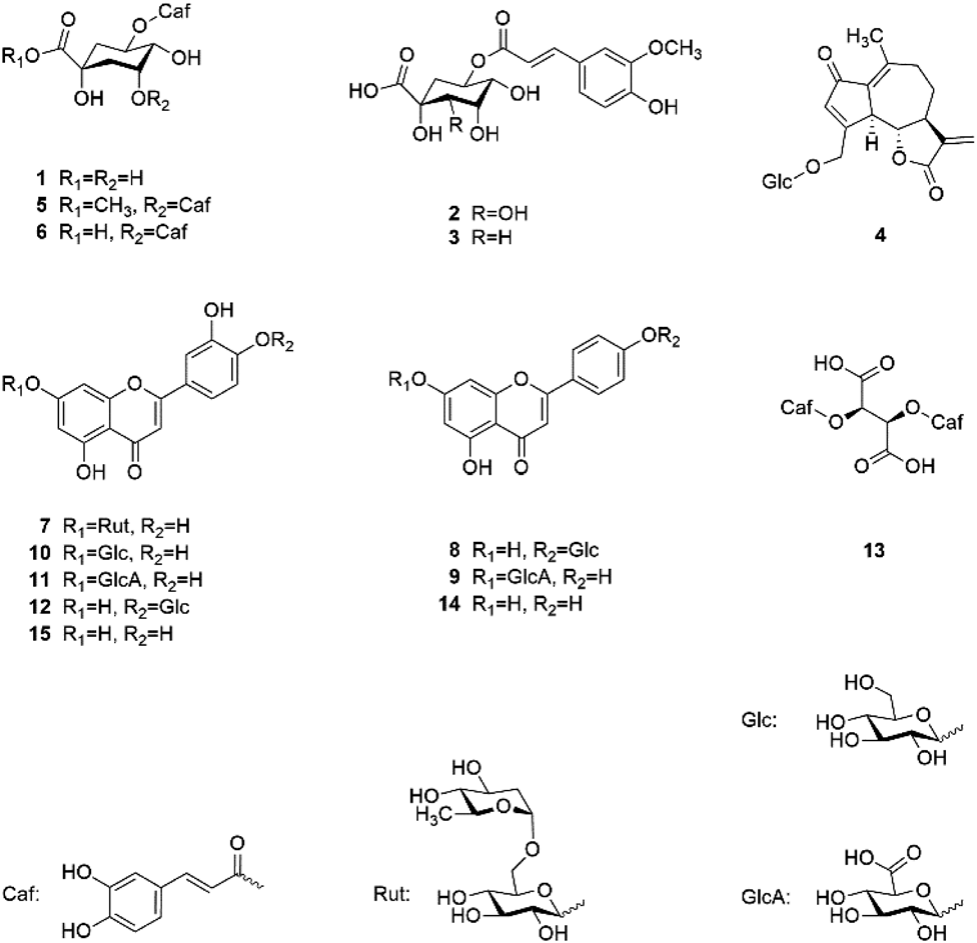
Chemical structures of isolated and identified compounds.

Fig. 1 shows the chemical structures of the 13 isolated and the 2 commercially obtained compounds.

### 2.5. Compound 2 – (1*R*,2*S*,3*S*,4*R*,5*R*)-1,2,3,4-tetrahydroxy-5-(((*E*)-4-hydroxy-3-methoxystyryl)oxy)cyclohexane-1-carboxylic acid

White powder; [*α*]_D_20 = −20.2° (*c* 0.5, MeOH); HRESIMS *m*/*z* 385.11213 [M + H]^+^ (calcd for C_17_H_21_O_10_, 385.11292) and *m*/*z* 407.09416 [M + Na]^+^ (calcd for C_17_H_20_O_10_Na, 407.09487); ^1^H NMR (400 MHz, DMSO-*d*_6_) *δ* 7.53 (1H, d, *J* = 15.6 Hz), 7.31 (1H, d, *J* = 1.8 Hz), 7.10 (1H, dd, *J* = 8.3 and 1.7 Hz), 6.80 (1H, d, *J* = 8.2 Hz), 6.45 (1H, d, *J* = 15.9 Hz), 5.14 (1H, td, *J* = 10.5 and 4.8 Hz), 3.89 (1H, t, *J* = 2.7 Hz), 3.82 (3H, s), 3.79 (1H, m), 3.54 (1H, dd, *J* = 9.8 and 1.8 Hz), 1.90 (1H, dd, *J* = 13.7 and 4.6 Hz), 1.73 (1H, t, *J* = 11.6 Hz). ^13^C NMR (400 MHz, DMSO-*d*_6_) *δ* 175.3, 166.5, 149.3, 148.0, 144.8, 125.7, 123.1, 115.5, 115.1, 111.1, 78.5, 75.4, 71.9, 70.4, 70.0, 55.7, 36.9.

### 2.6. Cytotoxicity assays

Myeloma cell lines (NCI-H929, OPM-2, U266) were incubated in cell culture media for 48 h with different concentrations of compounds. Induction of apoptosis was then measured by staining the cells with AnnexinV-fluorescein isothiocyanate and propidium iodide as described previously.^13^ Bortezomib (Eubio, Vienna, Austria) was used as positive control in different concentrations (5–20 nM) and a solvent control (DMSO) was always included. Assays were performed in triplicates at three different time points. The extent of non-apoptotic cells (AnnexinV/propidium iodide negativity) was calculated as percentage of viable cells in respect to the untreated control. Data are shown as mean percentage of viable cells and standard deviation (error bars). Myeloma cell lines were purchased from DSMZ (Braunschweig, Germany) and routinely fingerprinted and tested for mycoplasma negativity. Primary human foreskin fibroblasts were purchased from Promocell, Heidelberg, Germany. HS5 human bone marrow-derived stromal cells were purchased from ATCC (LGC Standards, Wesel, Germany). Peripheral blood mononuclear cells (PBMC) from three healthy donors were used after obtaining informed consent at the University Hospital Salzburg (ethics committee approval 415-E/1287/6-2011). These cells were subjected to Ficoll separation (Ficoll Paque™, VWR, Darmstadt, Germany) before use in cytotoxicity assays.

All cells were grown in RPMI-1640 media (Life Technologies, Paisley, UK) supplemented with 10% fetal calf serum (FCS; PAA, Linz, Austria), l-glutamine 100 μg mL^−1^ (Biochrom, Berlin Germany), and penicillin-streptomycin 100 U mL^−1^ (Applichem, Darmstadt, Germany). Adherent fibroblasts and HS5 stromal cells were seeded at a concentration of 1 × 10^4^ cells per well in 96-well plates the day before treatment in order to allow attachment. This media was then replaced by media containing the respective compounds at the indicated concentrations and incubated for 48 h before harvest and staining. Myeloma cells and PBMC were seeded in triplicates at a concentration of 1 × 10^5^ cells per well and incubated with the indicated compounds for 48 h.

## 3. Results and discussion

### 3.1. Activity-guided fractionation

**Fig. 2:**
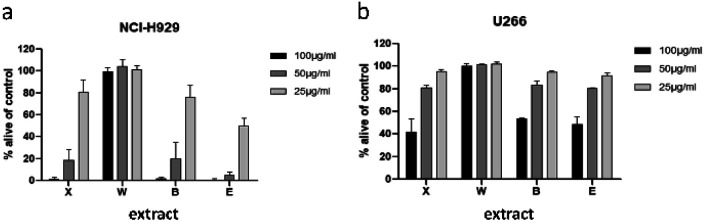
Viability of NCI-H929 (a) and U266 (b) cells after treatment with crude acetone 70% extract (X), and water (W), 1-butanol (B), or ethyl acetate (E) partitions. Viability was measured by flow cytometry (AnnexinV and propidium iodide negativity) and was calculated as percentage of untreated control.

To minimize the number of possible cytotoxic candidates, the crude acetone-water extract was partitioned between water and ethyl acetate and subsequently between water and *n*-butanol. As shown in Fig. 2, both the ethyl acetate and the *n-*butanol partitions were significantly decreasing the viability of NCI-H929 cell lines at concentrations of 25, 50 and 100 μg mL^−1^. Both partitions were also affecting U266 cells, but to a much lesser extent. Only the water layer did not show any effect against the two tested cell lines. Thus, it was decided to further fractionate the ethyl acetate as well as the *n-*butanol layer by vacuum liquid chromatography.

**Fig. 3:**
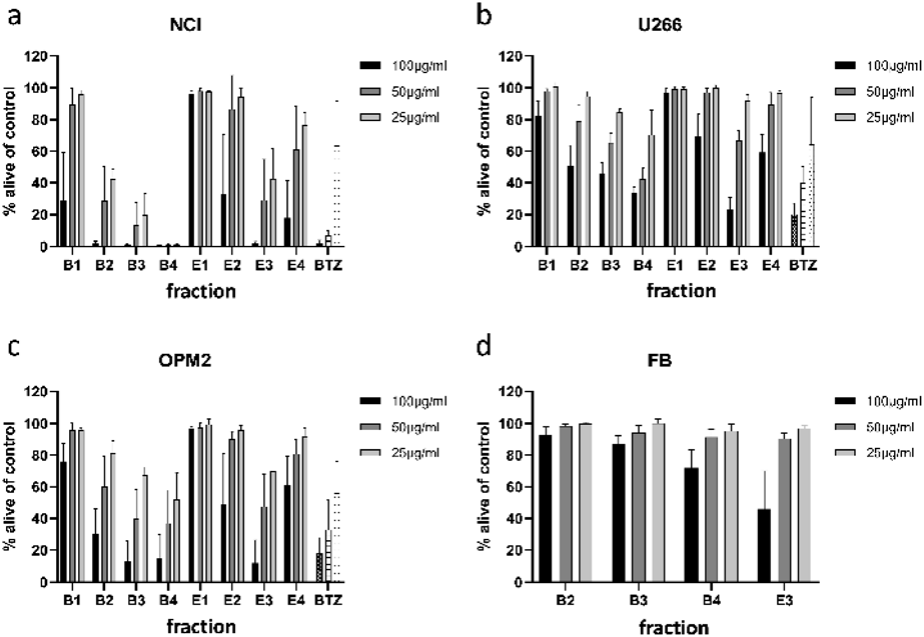
Viability of NCI-H929 (NCI) (a), U266 (b), OPM2 (c), and healthy fibroblast (d) cells after treatment with fractions obtained from 1-butanol (B1–B4) and ethyl acetate (E1–E4) partitions. Viability was measured by flow cytometry (AnnexinV and propidium iodide negativity) and was calculated as percentage of untreated control. BTZ (bortezomib) was used as positive control in concentrations of 20/10/5 nM.

For separation of the ethyl acetate partition, silica gel was chosen as stationary phase and organic solvents of increasing polarity to obtain four fractions (E1 to E4). The *n-*butanol partition was chromatographed using Diaion HP-20 material as stationary phase and different mixtures of water and methanol to obtain five fractions (B1 to B5). All fractions were subjected to cytotoxicity tests (Fig. 3a–c), however, fraction B5 could not be measured due to its intense dark brown colour. Still, the (only limited) cytotoxic potential of this fraction could be deduced from FACS experiments (data not shown). This time fractions were also examined for their effect on OPM2 cell lines and (if cytotoxicity was detected) on healthy fibroblast cells in order to see if the toxicity was of a general cytotoxic nature.

Of the four fractions obtained from the *n-*butanol partition, three fractions (B2 to B4) significantly affected NCI-H929 cells at all tested concentrations. Of the three fractions, B4 was the most effective, killing almost all treated cells at concentrations of 50 and 25 μg mL^−1^. The fractions obtained from the ethyl acetate partition were less effective. Only fraction E3 decreased NCI-H929 cells in a similar manner, being as effective as B2 but less effective as fractions B3 and B4. Fraction E3, moreover, showed the same activity pattern as fractions B3 and B4 against OPM2 cells and a similar overall activity as these fractions against the U266 cell line.

To find out if the cytotoxic effect of fractions B2, B3, B4, and E3 was resulting from general toxicity, their effect on healthy fibroblast cells was examined (Fig. 3d). For all four fractions, the effect on healthy fibroblast cells was much less pronounced than the effect on the studied cancer cell lines, indicating at least some degree of selectivity. Furthermore, the effect on healthy fibroblast cells was almost negligible at concentrations of 25 and 50 μg mL^−1^. Only at a concentration of 100 μg mL^−1^, a clear effect could be observed for two of the four tested fractions. Thus, fractions B2, B3, B4, and E3 were further analysed for their phytochemical composition.

### 3.2. Isolation and identification

Fraction B4, which showed the best overall activity against the tested cancer cell lines, was found to contain several flavonoid glycosides after LC-MS analysis. Their fragmentation pattern furthermore indicated these glycosides to derive from apigenin and luteolin scaffolds. Fractions B2 and B3 were also found to contain some of the mentioned flavonoids but showed additional signals indicating the presence of hydroxycinnamic acid derivatives. Fraction E3, in contrast, did not show any hydroxycinnamic acids but some flavonoid glycosides and the two aglycones apigenin and luteolin. There have been several reports on the cytotoxicity of apigenin and luteolin on different cancer entities (including myeloma cells).^16,19^ Thus, it was decided to obtain commercial samples of these two compounds and to focus on the isolation of the respective glycosides and the additional components in fractions B2 to B4.

Separation of fraction B4 resulted in the isolation of six flavone glycosides, which were identified as luteolin 7-*O*-β-d-rutinoside (7), apigenin 4′-*O*-β-d-glucoside (8), apigenin 7-*O*-β-d-glucuronide (9), luteolin 7-*O*-β-d-glucoside (10), luteolin 7-*O*-β-d-glucuronide (11), and luteolin 4′-*O*-β-d-glucoside (12) after LC-MS analysis and comparison of NMR spectra to literature data,^20–22^ as well as cichoric acid (13).^23^ Fraction B3 yielded one sesquiterpenoid, namely crepidiaside A (4),^24^ and five hydroxycinnamic acid derivatives, of which four were identified as chlorogenic acid (1), 5-feruloylquinic acid (3), 3,5-dicaffeoylquinic acid (6), and methyl 3,5-dicaffeoyl quinate (5).^25,26^ NMR data of all isolated compounds are given in Tables S1–S5.†

**Fig. 4:**
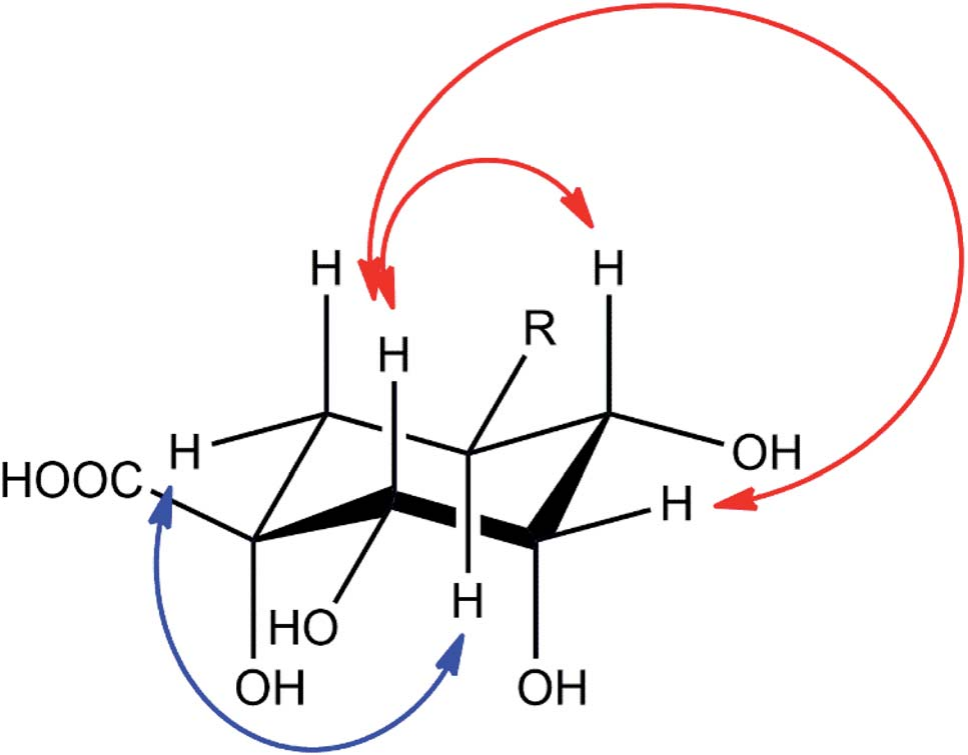
Important NOESY correlations of the H2- and H5-methine protons of compound 2, indicated by red (H2) and blue (H5) arrows, respectively.

Compound 2 showed a molecular mass of 384 Da and thus 30 Da more than chlorogenic acid (1), indicating both an additional hydroxylation and methylation. NMR spectra showed that the compound is a derivative of 5-feruloylquinic acid (3), lacking the signals of one of the two methylene groups in the quinic acid ring and showing a methine signal instead (Fig. S1–S6†). This signal was shifted into the region of the other hydroxymethine signals, thus confirming additional hydroxylation of the molecule. The decreased shift values of the methine proton in position 3 furthermore suggested that the hydroxy-group is located in position 2 of the molecule. This assumption was also deduced from NOESY experiments, which showed coupling of the H5-proton with the (remaining) methylene group in position 6 (Fig. 4). NOESY spectra furthermore revealed strong coupling of the H2-methine proton with the β-oriented H4-proton and lower coupling to the equatorial H3-proton. In contrast, no coupling of the H2-proton to the α-oriented H5-proton was observed. Therefore, the hydroxy-group had to be substituted in equatorial position and the compound to be (1*R*,2*S*,3*S*,4*R*,5*R*)-1,2,3,4-tetrahydroxy-5-(((*E*)-4-hydroxy-3-methoxystyryl)oxy)cyclohexane-1-carboxylic acid or 5-feruloyl-2α-hydroxyquinic acid, respectively.

### 3.3. Cytotoxicity measurements

**Fig. 5:**
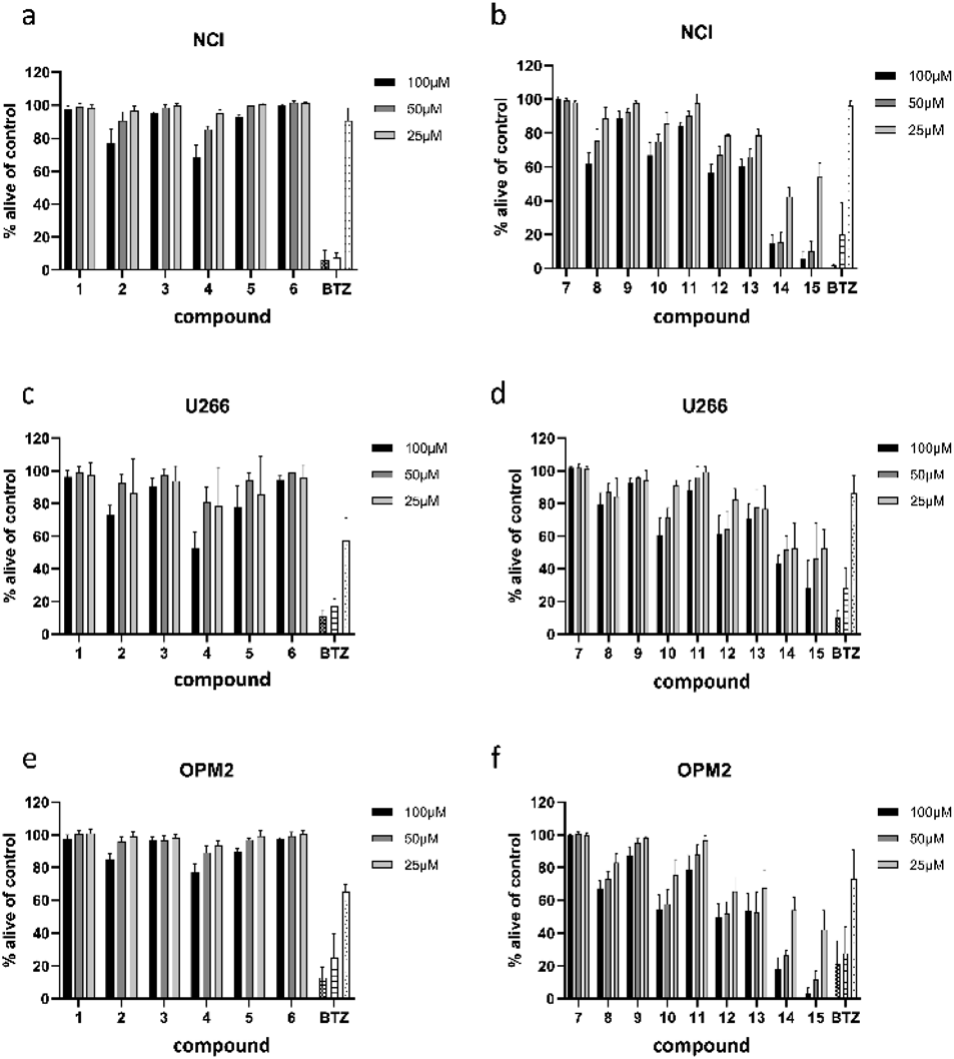
Viability of NCI-H929 (NCI) (a and b), U266 (c and d), and OPM2 (e and f) cells after treatment with compounds present in *L. saxatilis*. Viability was measured by flow cytometry (AnnexinV and propidium iodide negativity) and was calculated as percentage of untreated control. BTZ (bortezomib) was used as positive control in concentrations of 20/10/5 nM.

All 13 isolated compounds and the 2 commercially obtained flavonoids apigenin (14) and luteolin (15) were tested for their effect on NCI-H929, U266, and OPM-2 myeloma cell lines (Fig. 5). Of the six tested hydroxycinnamic acid derivatives, chlorogenic acid (1), 5-feruoylquinic acid (3), and 3,5-dicaffeoylquinic acid (6) showed no activity. However, methyl 3,5-dicaffeoylquinate (5) and the new natural product 5-feruoyl-2α-hydroxyquinic acid (2) at least exhibited weak to moderate effects at the highest concentration tested. In contrast, cichoric acid (13) was decreasing the viability of the tested cell lines at all measured concentrations. Here, the strongest effect was observed against OPM-2 cells, where viability was decreased by approximately 50%.

Crepidiaside A (4), which was the only sesquiterpenoid found in *L*. *saxatilis*, was moderately active at a concentration of 100 μM and still slightly active at 50 μM. In contrast to cichoric acid (13), crepidiaside had higher impact on U266 cells than on the other two cell lines.

Altogether eight flavonoids were investigated for their effect on myeloma cells. Out of these, compounds 7, 9, and 11, did not display significant activity on any of the cell lines. Compounds 8, 10, and 12, instead, showed moderate effects at higher concentrations and weak effects at 25 μM. On the contrary, apigenin (14) and luteolin (15) exhibited strong effects on NCI and OPM-2 cells at concentrations of 50 and 100 μM and were still moderately active at 25 μM. Moreover, both compounds showed moderate effects on U266 cells over the whole concentration range.

### 3.4. Determination of selectivity

**Fig. 6:**
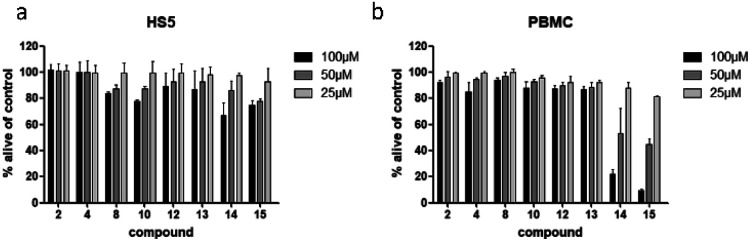
Viability of human bone marrow derived stroma cell line (HS5) (a), and peripheral mononuclear blood cells (PBMC) (b) after treatment with compounds present in *L. saxatilis*. Viability was measured by flow cytometry (AnnexinV and propidium iodide negativity) and was calculated as percentage of untreated control.

To examine if the identified cytotoxic constituents display selectivity for myeloma cells, we tested the most active compounds 2, 4, 8, 10, and 12–15 against two different non-cancerous cell types (Fig. 6). First, we assessed the compounds' activity against human bone marrow derived stroma cells (HS5) that are found in close proximity to the myeloma cancer cells in the bone marrow. Secondly, we measured their effects on the viability of peripheral blood mononuclear cells (PBMC) as a counterpart to the malignant plasma cells.

All eight investigated compounds clearly showed selectivity for myeloma cells *versus* human stroma cell line HS5 (Fig. 6a) and with few exceptions against healthy blood cells (Fig. 6b). Compounds 2 and 4, which showed weak to moderate activity against all myeloma cell lines at a concentration of 100 μM, left HS5 cells completely unaffected and only slightly decreased viability of PBMC at the same concentration. Flavone glycosides 8, 10, and 12 showed pronounced selectivity for myeloma cells as well. Interestingly, especially for compound 10, stroma cells appeared to be more susceptible than PBMC, however, myeloma cells were more affected than stroma cells. The same accounts for cichoric acid (13), which showed higher effects against all three myeloma cell lines than against healthy PBMC and HS5 cells. The most cytotoxic substances in this screen were apigenin (14) and luteolin (15). All tested cells displayed enhanced apoptosis with a clear preference of cell death in blood-borne cells. Here, myeloma cells were affected to a much higher extend than healthy PBMC.

Apart from demonstrating selectivity of the tested compounds for myeloma cells, the results for apigenin (14) and luteolin (15) on healthy PBMC furthermore corroborate the before obtained results against healthy fibroblast cells, which showed a higher general toxicity for the fraction in which these two compounds were enriched.

### 3.5. Structure–activity relationships

Hydroxycinnamic acid derivatives are known to exhibit various biological effects, such as antioxidative and antiviral activities, as well as tyrosinase, α-glucosidase, and PTP1B inhibitory effects.^25–27^ However, against myeloma cell lines so far only caffeic acid phenethyl ester (CAPE) was found to induce apoptosis, which is achieved by activation of caspase-3 and corresponding cleavage of poly(ADP-ribose)polymerase.^28^ The observed activity of cichoric acid (13) in our study thus is reasonable. In contrast to the other isolated hydroxycinnamic acid derivatives (1–3, 5, 6), cichoric acid is lacking the quinic acid ring and same as CAPE shows a closer connection of the two arylalkylic substructures than dicaffeoylquinic acids 5 and 6. Furthermore, cichoric acid (13) is less polar than the other hydroxycinnamic acids investigated in this study, missing the additional hydroxyl-groups of the quinic acid ring, most present in monoesterified compounds 1–3.

Increased polarity might also be responsible for the comparably low activity of the glycosylated sesquiterpenoid crepidiaside A (4). Several sesquiterpenes have been reported to have an impact on myeloma cells *in vitro*, such as cnicin,^18^ parthenolide,^29^ alantolactone,^30^ bingelovine,^31^ 8-hydrocalamenene,^32^ or 6-*O*-angeloylplenolin.^33^ Most of these compounds show a lactone ring and/or the characteristic exocyclic methylene group, which is known to be cytotoxic by alkylation. Crepidiaside A (4) shows both of these features, but in contrast to the compounds described above, it does have a sugar moiety, which might be a reason for its limited activity against myeloma cells.

The group of investigated flavonoids allows the most detailed discussion of their structure–activity relationship. Not only because of the two different flavonoid scaffolds apigenin (14) and luteolin (15), but because of the different sugar moieties and their positions in the molecule. When looking at the results obtained for luteolin 7-*O*-β-d-rutinoside (7) it is evident that a disaccharidic moiety results in a complete loss of activity compared to the aglycone luteolin (15) or its monoglycosidic derivatives (10–12). Furthermore, substitution with glucuronic acid instead of glucose decreases the activity, as observed by the lower activity of luteolin 7-*O*-β-d-glucuronide (11) when compared to luteolin 7-*O*-β-d-glucoside (10), as well as the reduced activity of apigenin 7-*O*-β-d-glucuronide (9). Also, the position of glycosylation seems to have (a slight) impact on the activity, showing a somewhat stronger effect for luteolin 4′-*O*-β-d-glucoside (12) than for its 7-*O*-glycosylated counterpart (10). With regard to the flavonoid scaffold, apigenin type flavonoids exhibit a lesser impact on myeloma cells than luteolin-type flavonoids, as demonstrated by the lower effect of apigenin 4′-*O*-β-d-glucoside (8) in comparison to luteolin 4′-*O*-β-d-glucoside (12), as well as the lower activity of apigenin (14) compared to luteolin (15). Still, both aglycones were far more active than any of their glycosylated forms and (because of their good availability) have been subject of various pharmacological and toxicological studies.

Both apigenin (14) and luteolin (15) together with the related flavonoid chrysin (which is lacking both the 3′- and the 4′-hydroxy-group) were found to inhibit proteasome catalytic activities.^16^ A follow-up study investigated the anti-proteasome effect of baicalein and scutellarin, which show additional hydroxylation in position 6 when compared to chrysin and apigenin (14), respectively.^34^ Because both compounds were inactive, it was concluded that the 5,7-dihydroxylation pattern of chrysin, apigenin (14), and luteolin (15), is essential for the cytotoxic activity. The mentioned compounds were also part of a study on the anti-leukemic activity of *Scutellaria orientalis* extracts, which contain apigenin (14), baicalein, chrysin, luteolin (15), and wogonin, another flavone-type aglycone.^35^ Interestingly, a methanolic extract, which also contained several flavonoid glycosides (and not the more apolar and thus aglycone-rich extracts) turned out to be most active. This is in line with our own study, where the activity of the crude acetone (70%) extract (X, Fig. 2) was relatively high in comparison to the ethyl acetate (E) and *n*-butanol (B) partitions, in which either flavone alycones (E) or the respective glycosides (B) were enriched. Another point often discussed in phytopharmacology is the fact that the sum of many similar components contributes to the activity by having at the same time a lower toxic effect. This can also be concluded from the analysis of the different fractions on myeloma cell lines (Fig. 3a–c) and healthy, primary fibroblast cells (Fig. 3d). Fractions B3 and B4 were containing the slightly and moderately active hydroxycinnamic acids and flavonoid glycosides, but still exhibited comparable effects as fraction E3, which had high amounts of apigenin (14) and luteolin (15). At the same time B3 and B4 showed only weak toxicity against healthy fibroblast cells, while the flavone-rich fraction E3 was decreasing the viability of these cells by approximately 50% at the highest concentration.

Since apigenin (14) and luteolin (15) (and chrysin) have been shown to inhibit proteasomal activity and because survival of myeloma cells depends crucially on a functional proteasome, the synergistic and at the same time less toxic effect of many similar components observed in our study could be a great alternative. Here, future studies will reveal if *e.g.* combining lower doses of the standard myeloma therapy (and proteasome inhibitor) bortezomib with the active components in our studies could achieve the same therapeutic effects and also cause less side effects. This is even more interesting, as apigenin (14) was recently suggested as a general cancer medication^36^ and luteolin (15) has been found to suppress tumour metastasis in breast and colorectal cancer cells.^37,38^

## 4 Conclusions

In the present study, 15 natural products present in the aerial parts of *L. saxatilis* were investigated for their cytotoxic effects against different myeloma cell lines. The number of similar components of two important compound classes (flavonoids and hydroxycinnamic acids) allowed a detailed discussion of their structure–activity relationships. Our study, furthermore, confirmed the already known cytotoxic potential of apigenin (14) and luteolin (15) for additional myeloma cell lines and revealed antimyeloma properties for some of their glycosides as well as for cichoric acid (13). Moreover, our results suggested that the general toxicity was reduced (and the activity against myeloma cells maintained) by combining several similar components with originally lower activity.

Another highlight of this study is the isolation of a new hydroxycinnamic acid derivative, namely 5-feruloyl-2α-hydroxyquinic acid (2). Though, the compound showed only moderate cytotoxic effects, the occurrence of hydroxyquinic acids in nature is uncommon and has only been reported once in literature.^39^ But also, the identification of crepidiaside A (4) is of (taxonomic) importance. Within the genus *Leontodon* the occurrence of hypocretenolides was so far restricted to the section *Leontodon*, while other sections of the genus *Leontodon* were containing other types of sesquiterpenoids.^40^ Thus, the present study shows the first report of a hypocretenolide in the section *Thrincia*.

## Conflicts of interest

There are no conflicts to declare.

## Supplementary Material

RA-011-D0RA10973H-s001
