# Supplementary material for: Cytotoxic constituents and a new hydroxycinnamic acid derivative from Leontodon saxatilis (Asteraceae, Cichorieae)
Source: RSC Adv. 2021 Mar 10;11(18):10489–96. doi: 10.1039/d0ra10973h (PMC8695733; doi:10.1039/d0ra10973h)
Supplement: RA-011-D0RA10973H-s001 [file RA-011-D0RA10973H-s001.pdf]

Electronic Supplementary Material (ESI) for RSC Advances.
This journal is © The Royal Society of Chemistry 2021

Supporting information

Cytotoxic constituents and a new hydroxycinnamic acid derivative from *Leontodon saxatilis* (Asteraceae, Cichorieae)

Serhat Sezai Çiçek,[*a] Johanna Willer,[a] Francesca Preziuso,[ab] Frank Sönnichsen,[c] Richard Greil,[de] Ulrich Girreser,[f] Christian Zidorn [a] and Karin Jöhrer [d]

a. Department of Pharmaceutical Biology, Kiel University, Gutenbergstraße 76, 24118 Kiel, Germany.

b. Department of Pharmacy, University "G. d'Annunzio" of Chieti-Pescara, Via dei Verstini 31, 66100 Chieti Scalo (CH), Italy.

c. Otto Diels Institute for Organic Chemistry, Kiel University, Otto-Hahn-Platz 4, Kiel Germany.

d. Tyrolean Cancer Research Institute, Innrain 66, 6020 Innsbruck, Austria.

e. Paracelsus Medical University Salzburg, Department of Internal Medicine III, Salzburg Cancer Research Institute-Laboratory for Immunological and Molecular Cancer Research, Müllner Hauptstraße 48, 5020 Salburg, Austria.

f. Department of Pharmaceutical and Medicinal Chemistry, Kiel University, Gutenbergstraße 76, 24118 Kiel, Germany.

* Corresponding author: scicek@pharmazie.uni-kiel.de

Fig. S1:

^{1}H NMR spectrum of compound **2** measured in DMSO-d_6

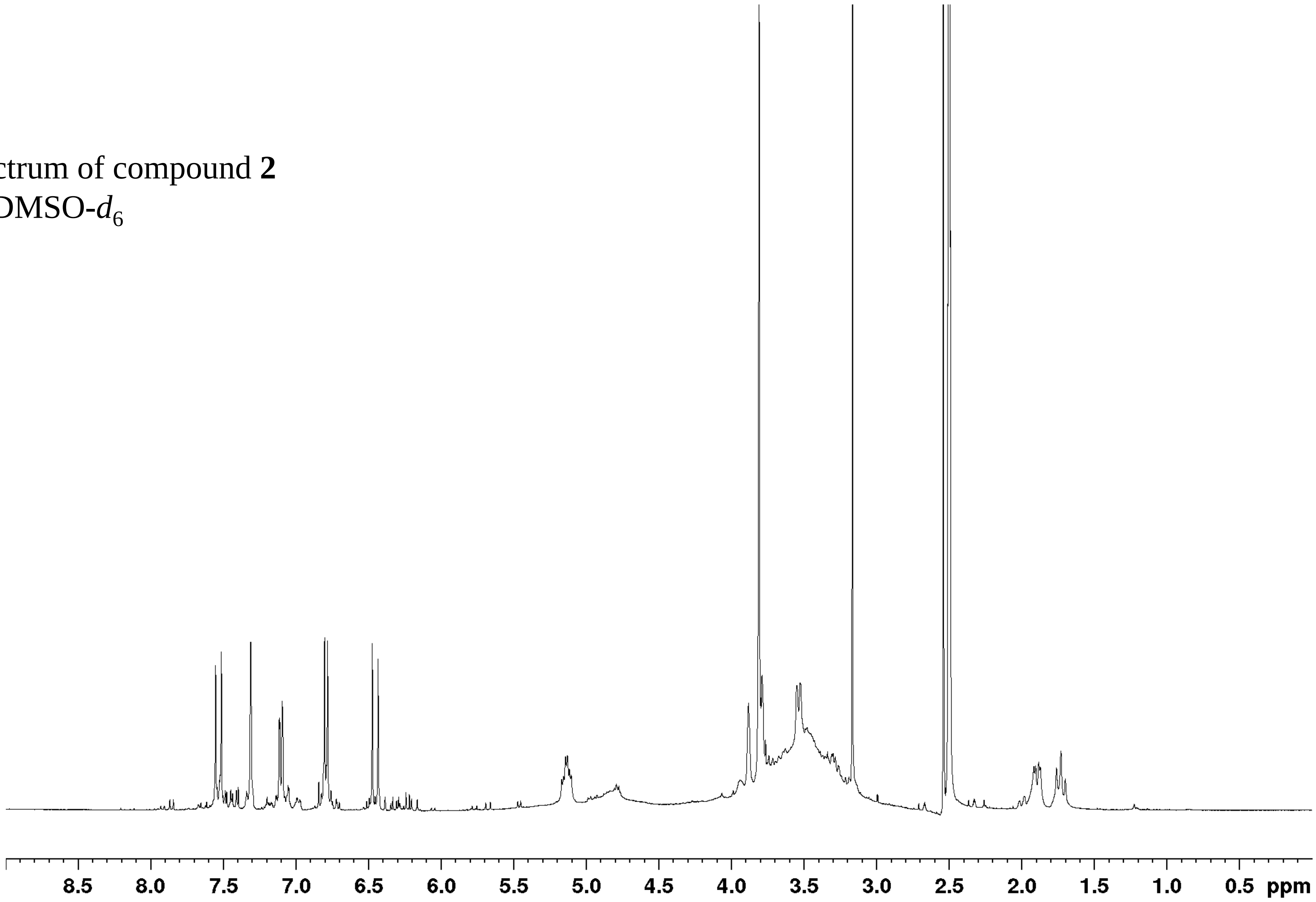

Fig. S2:

^{13}C NMR spectrum of compound **2** measured in DMSO-d_6

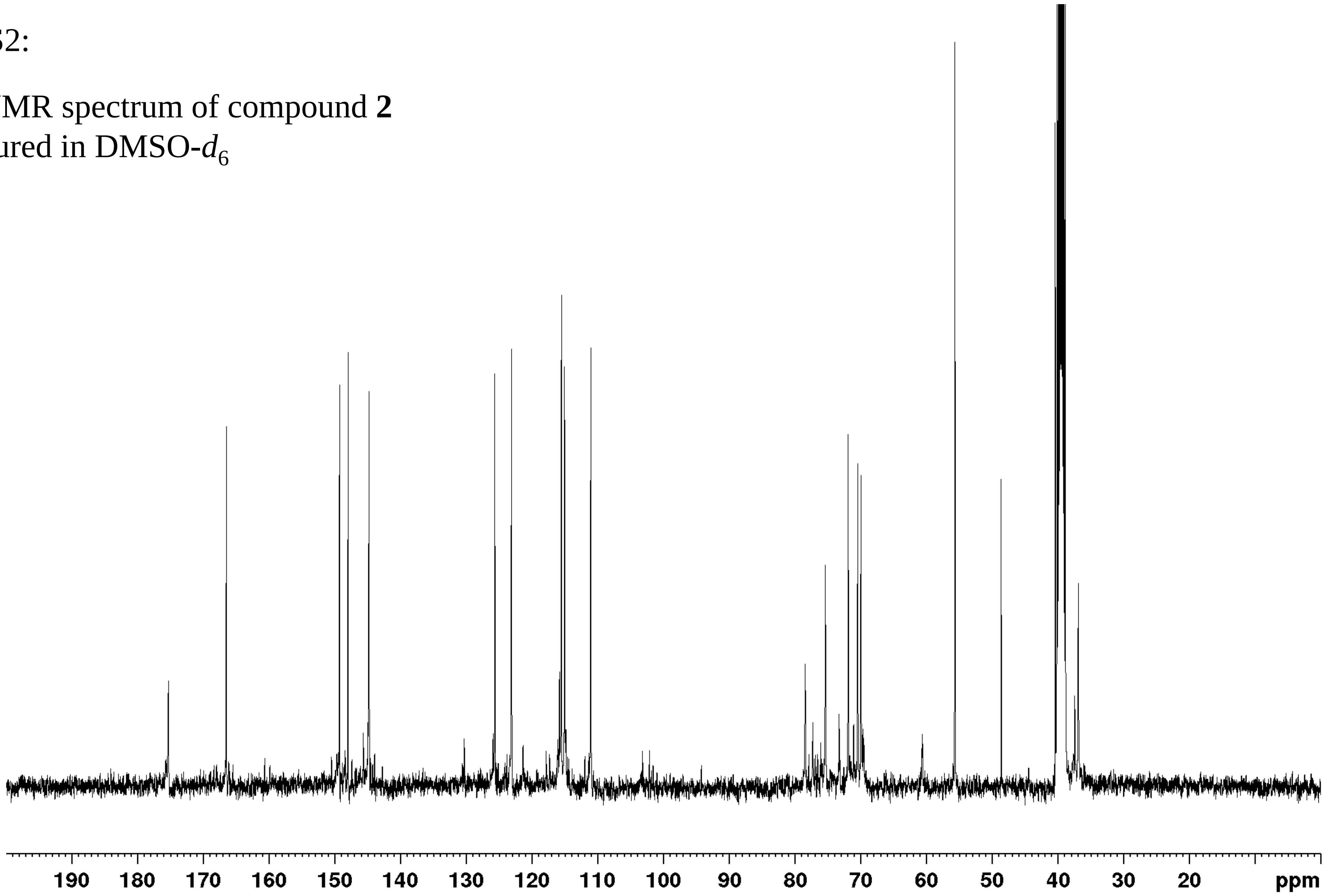

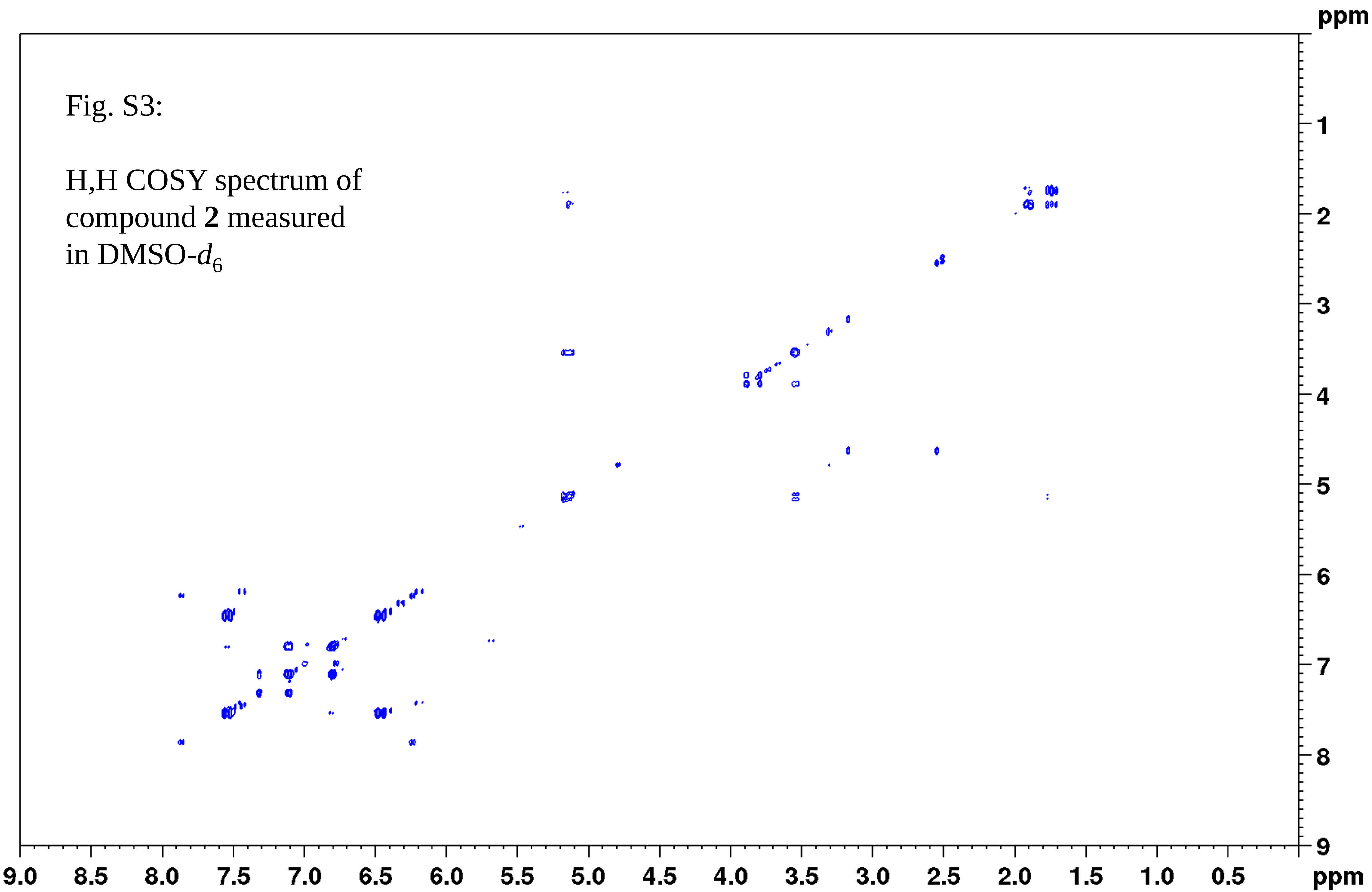
Fig. S3:
H,H COSY spectrum of compound **2** measured in DMSO-d_6
ppm
1
2
3
4
5
6
7
8
9
9.0
8.5
8.0
7.5
7.0
6.5
6.0
5.5
5.0
4.5
4.0
3.5
3.0
2.5
2.0
1.5
1.0
0.5
ppm

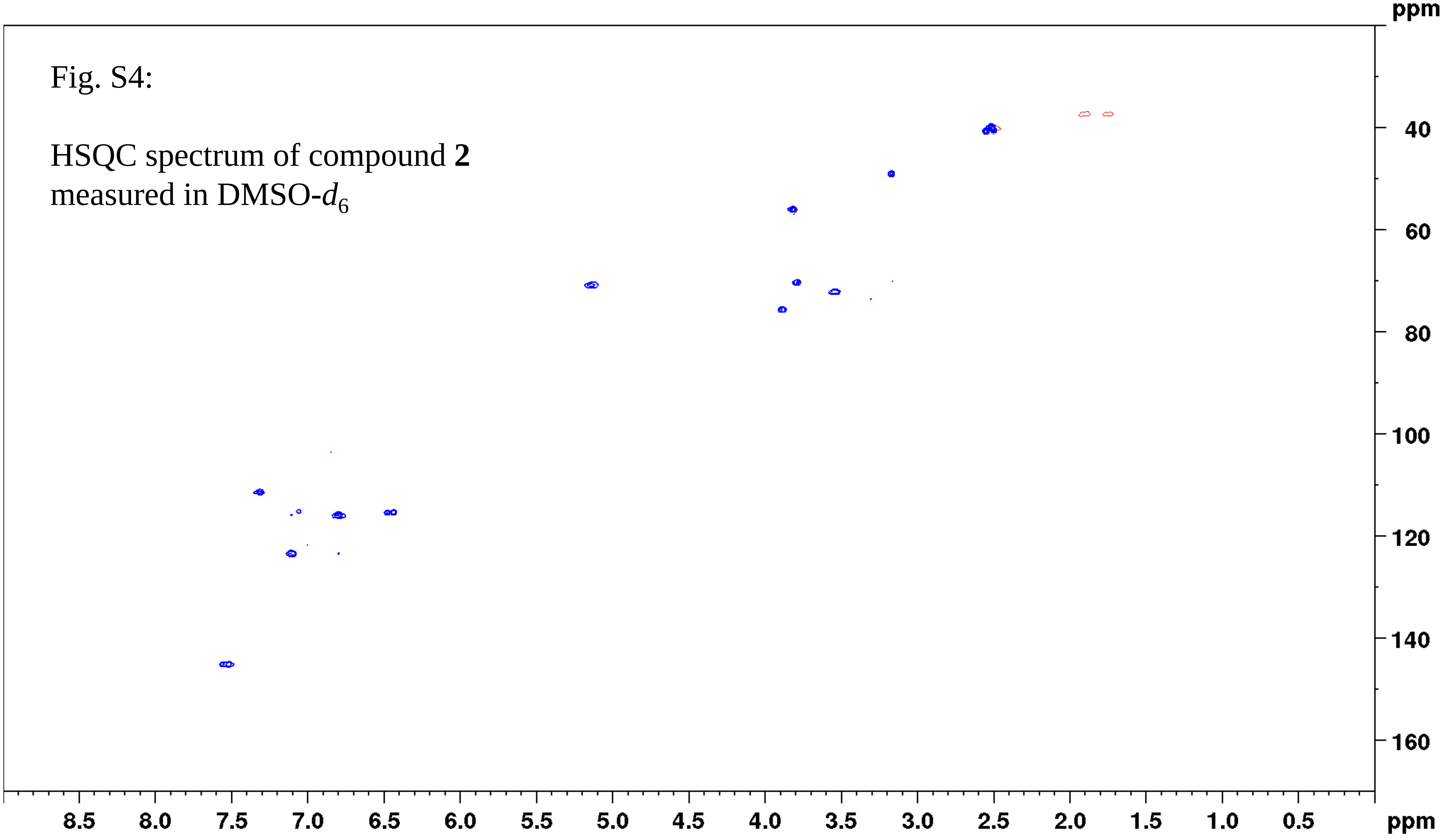
Fig. S4:
HSQC spectrum of compound **2** measured in DMSO-d_6
ppm
40
60
80
100
120
140
160
8.5
8.0
7.5
7.0
6.5
6.0
5.5
5.0
4.5
4.0
3.5
3.0
2.5
2.0
1.5
1.0
0.5
ppm

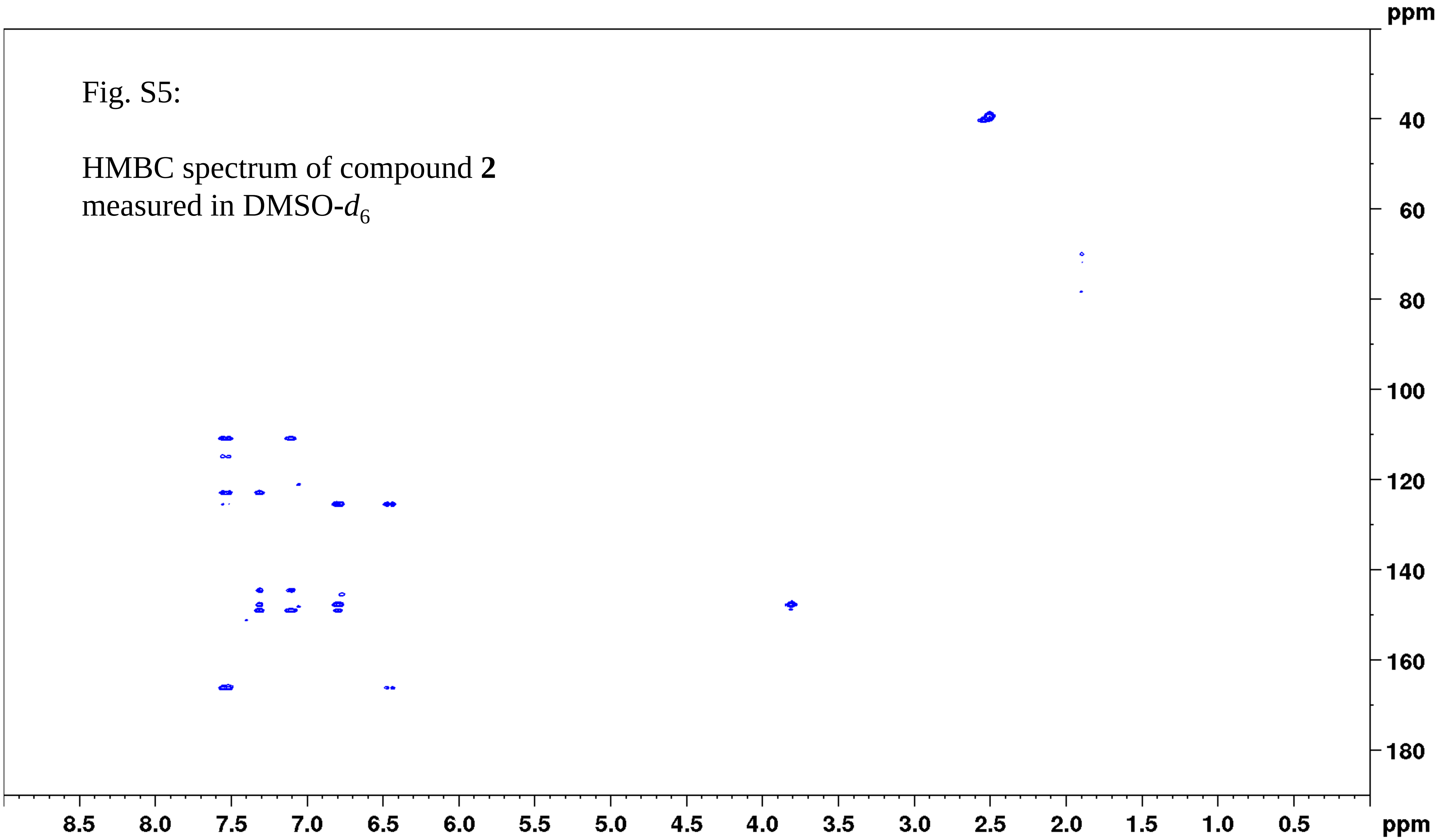

Fig. S5:

HMBC spectrum of compound **2** measured in DMSO-d_6

Fig. S6:

NOESY spectrum of compound **2** measured in DMSO-d_6

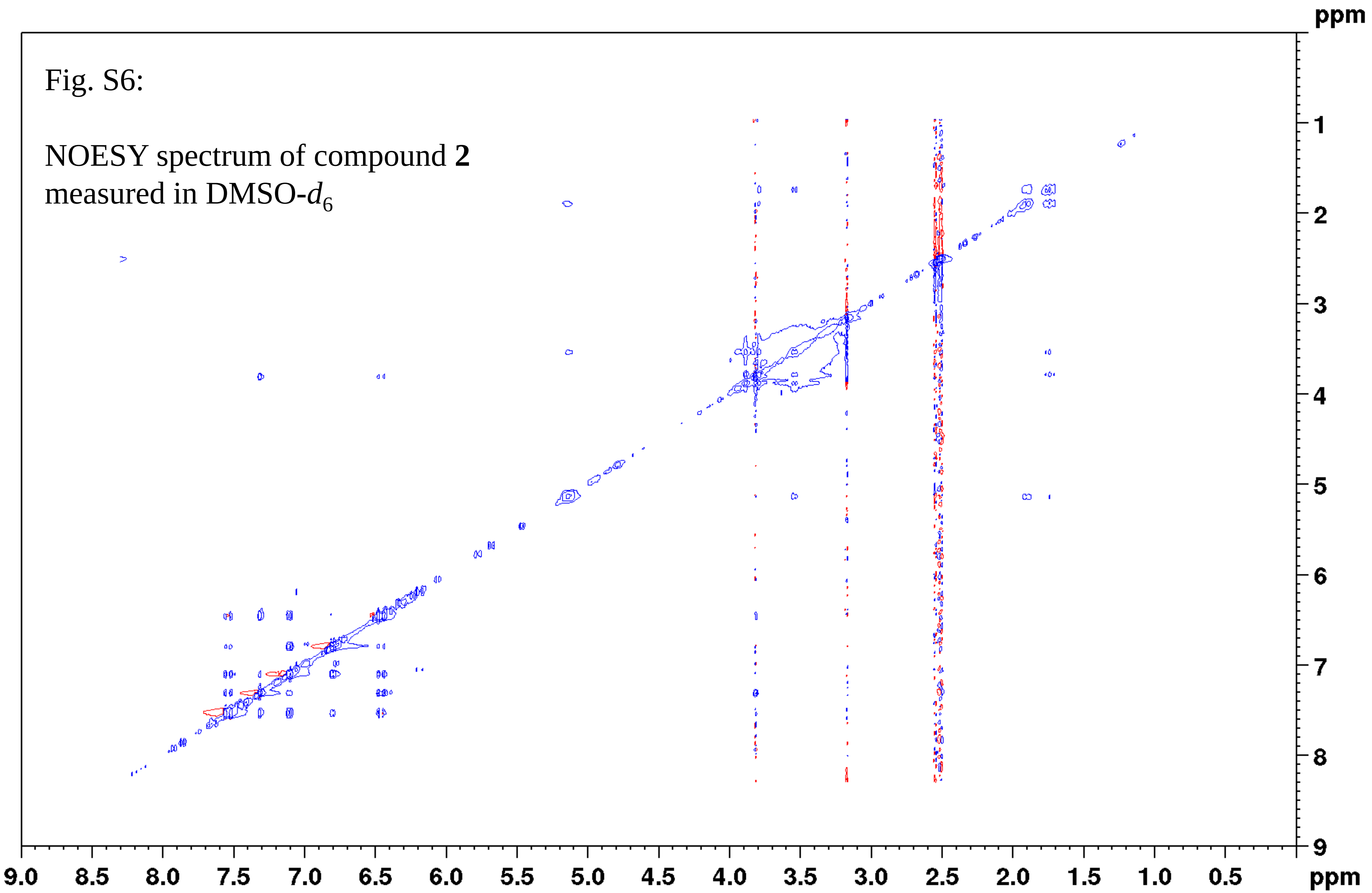

Table S1

^{1}H NMR data for compounds **1**, **2**, **3**, **5**, **6,** and **13** (400 MHz, δ in ppm, J in Hz). [a] MeOH-d_4 (3.31 ppm), [b] DMSO-d_6 (2.50 ppm)

Position	**1** [a]	**2** [a]	**3** [a]	**5** [a]	**6** [a]	**13** [b]
2	2.02 (m)<br>2.16 (m)	4.15 (m)	2.02 (m)<br>2.16 (m)	2.16 (m)<br>2.34 (m)	2.17 (m)<br>2.32 (m)	5.50 (s)
3	4.16 (s)	4.04 (s)	4.16 (s)	5.40 (m)	5.41 (m)	5.50 (s)
4	3.71 (m)	3.74 (m)	3.70 (m)	3.99 (m)	3.99 (m)	
5	5.37 (m)	5.34 (s)	5.40 (s)	5.31 (m)	5.32 (m)	
6	2.04 (m)<br>2.19 (m)	1.93 (m)<br>2.21 (m)	2.03 (m)<br>2.17 (m)	2.19 (m)<br>2.30 (m)	2.21 (m)<br>2.30 (m)	
2’	7.05 (s)	7.18 (s)	7.20 (s)	7.06 (m)	7.07 (m)	7.05 (s)
5’	6.78 (d, 8.0)	6.81 (d, 8.4)	6.81 (d, 7.8)	6.79 (d, 8.5)	6.79 (d, 8.5)	6.75 (d, 8.3)
6’	6.95 (d, 8.5)	7.07 (d, 7.8)	7.08 (d, 7.8)	6.95 (m)	6.98 (m)	7.01 (d, 8.3)
7’	7.56 (d, 15.9)	7.63 (d, 16.4)	7.64 (d, 16.0)	7.62 (d, 16.0)	7.62 (d, 16.0)	7.49 (d, 16.0)
8’	6.29 (d, 16.0)	6.38 (d, 16.4)	6.40 (d, 15.8)	6.35 (d, 16.0)	6.35 (d, 16.0)	6.28 (d, 16.0)
2’’				7.06 (m)	7.07 (m)	7.01 (d, 8.3)
5’’				6.78 (d, 8.5)	6.78 (d, 8.5)	6.75 (d, 8.3)
6’’				6.95 (m)	6.96 (m)	7.05 (s)
7’’				7.56 (d, 16.0)	7.56 (d, 16.0)	7.49 (d, 16.0)
8’’				6.23 (d, 16.0)	6.22 (d, 16.0)	6.28 (d, 16.0)
$COOCH_3$				3.65 (s)		
OCH_3		3.89 (s)	3.89 (s)			

Table S2

^{13}C NMR data for compounds **1**, **2**, **3**, **5**, **6**, and **13** (100 MHz, δ in ppm). [a] MeOH-d_4 (49.0 ppm), [b] DMSO-d_6 (39.5 ppm), n.o. means not observed

Position	**1** [a]	**2** [a]	**3** [a]	**5** [a]	**6** [a]	**13** [b]
1	n.o.	76.7	n.o.	74.8	74.8	168.4
2	38.8	71.6	37.1	35.9	35.9	70.6
3	72.7	73.6	71.4	72.0	72.2	70.6
4	74.6	71.5	73.2	69.8	69.8	168.4
5	72.5	37.7	70.9	72.3	72.3	
6	40.2	127.8	38.7	36.8	36.8	
1’	128.0	111.7	126.6	126.6	126.6	125.4
2’	115.2	149.3	110.0	115.3	115.3	115.1
3’	147.2	150.6	148.3	148.3	148.3	148.7
4’	149.8	116.4	149.1	149.1	149.1	145.7
5’	116.6	124.1	114.9	116.5	116.7	115.9
6’	123.0	146.9	122.4	123.1	123.2	121.5
7’	147.0	115.7	145.1	147.4	147.4	145.9
8’	115.6	168.9	114.3	115.5	115.6	113.5
9’	169.1		169.2	169.2	169.2	165.9
1’’				126.6	126.6	125.4
2’’				115.3	115.3	115.1
3’’				148.3	148.3	148.7
4’’				149.1	149.1	145.7
5’’				116.5	116.7	115.9
6’’				123.1	123.2	121.5
7’’				147.6	147.6	145.9
8’’				115.0	115.0	113.5
9’’				169.2	169.2	165.9
COO	n.o.	n.o.	n.o.	175.8	175.8	
OCH_3		56.4	54.8	53.2		

Table S3

^{1}H and ^{13}C NMR data for compound **4** in methanol-d_4 (400 MHz, δ in ppm, J in Hz).

Position	^{1}H NMR	^{13}C NMR
1		132.7
2		197.9
3	6.58 (d, 1.2)	134.8
4		171.8
5	3.86 (m)	51.3
6	3.64 (t, 10.2)	85.9
7	3.03 (m)	53.7
	1.44 (m)	
8	2.26 (m)	25.4
	2.43 (m)	
9	2.62 (br t, 13.5)	38.3
10		156.3
11		140.7
12		171.2
	5.56 (d, 3.2)	
13	6.10 (d, 3.3)	119.4
14	2.44 (s)	22.2
	4.18 (d, 12.5)	
15	4.80 (br d, 17.2)	69.8
1’	4.40 (d, 7.6)	104.3
2’	3.26 (dd, 7.8, 9.0)	75.3
3’	3.31 (m)	78.2
4’	3.33 (m)	71.7
5’	3.38 (m)	78.2
	3.69 (dd, 5.0, 12.0)	
6’	3.90 (m)	62.8

Table S4

^{1}H NMR data for compounds **7–12** in DMSO-d_6 (400 MHz, δ in ppm). Ref. DMSO: 2.50 ppm

Position	**7**	**8**	**9**	**10**	**11**	**12**
3	6.75 (s)	6.91 (s)	6.88 (s)	6.76 (s)	6.76 (s)	6.83 (s)
6	6.44 (d, 2.2)	6.21 (d, 2.1)	6.47 (d, 2.2)	6.44 (d, 2.2)	6.46 (d, 2.1)	6.20 (d, 2.1)
8	6.74 (d, 2.2)	6.52 (d, 2.1)	6.86 (d, 2.2)	6.79 (d, 2.2)	6.82 (d, 2.1)	6.50 (d, 2.1)
2’	7.43 (s)	8.05 (d, 8.9)	7.96 (d, 8.7)	7.43 (s)	7.42 (s)	7.50 (s)
3’		7.19 (d, 8.9)	6.94 (d, 8.7)			
5’	6.91 (d, 8.5)	7.19 (d, 8.9)	6.94 (d, 8.7)	6.91 (d, 8.3)	6.91 (d, 8.3)	7.23 (d, 8.6)
6’	7.45 (d, 8.5)	8.05 (d, 8.9)	7.96 (d, 8.7)	7.45 (d, 8.3)	7.45 (d, 8.3)	7.52 (d, 8.6)
1’’	5.08 (d, 7.3)	5.03 (d, 7.3)	5.28 (d, 7.3)	5.09 (d, 7.3)	5.29 (d, 7.3)	4.89 (d, 7.5)
2’’	3.28 (m)	3.28 (m)	3.31 (m)	3.28 (m)	3.31 (m)	3.33 (m)
3’’	3.30 (m)	3.29 (m)	3.32 (m)	3.30 (m)	3.33 (m)	3.32 (m)
4’’	3.17 (m)	3.18 (m)	3.40 (m)	3.18 (m)	3.39 (m)	3.18 (m)
5’’	3.60 (m)	3.41 (m)	4.05 (d, 9.5)	3.45 (m)	4.04 (d, 9.4)	3.39 (m)
6’’	3.46 (m)	3.48 (m)		3.48 (m)		3.48 (m)
	3.84 (dd, 1.6, 11.5)	3.70 (dd, 4.6, 11.2)		3.71 (dd, 4.8, 10.9)		3.72 (dd, 4.0, 11.1)
1’’’	4.54 (d, 1.1)					
2’’’	3.46 (m)					
3’’’	3.65 (m)					
4’’’	3.14 (m)					
5’’’	3.41 (m)					
6’’’	1.07 (d, 6.2)					

Table S5

^{13}C NMR data for compounds **7–12** in DMSO-d_6 (100 MHz, δ in ppm). Ref. DMSO: 39.5 ppm

Position	**7**	**8**	**9**	**10**	**11**	**12**
2	163.0	163.0	164.3	161.8	164.5	163.4
3	103.3	103.7	103.2	108.7	103.3	103.9
4	182.0	181.8	182.0	177.4	181.7	181.6
5	161.3	161.5	161.2	163.1	161.2	161.6
6	99.6	98.8	99.3	104.8	99.3	98.8
7	164.6	164.5	162.5	159.1	162.4	164.3
8	94.8	94.0	94.7	98.6	94.7	94.0
9	157.0	157.4	157.0	158.8	156.9	157.3
10	105.4	103.8	105.4	106.1	105.5	104.0
1’	121.5	121.0	121.0	122.0	119.3	124.4
2’	113.6	128.2	128.7	113.6	113.4	113.7
3’	145.8	116.5	116.0	146.1	145.6	146.8
4’	150.0	160.2	161.3	149.7	149.9	148.4
5’	116.1	116.5	116.0	116.4	116.1	115.9
6’	119.3	128.2	128.7	119.0	119.2	118.4
1’’	99.9	99.8	99.1	105.0	99.0	101.0
2’’	73.1	73.1	72.9	74.1	72.9	73.1
3’’	76.3	76.4	75.7	78.0	75.7	75.9
4’’	69.5	69.5	71.2	70.2	71.3	69.7
5’’	75.6	77.1	75.4	76.1	75.4	77.3
6’’	66.0	60.5	170.2	61.3	170.2	60.7
1’’’	100.6					
2’’’	70.8					
3’’’	70.4					
4’’’	72.1					
5’’’	68.4					
6’’’	17.8					
